# Genome Sequence of a Facklamia hominis Isolate from a Patient with Urosepsis

**DOI:** 10.1128/MRA.00100-19

**Published:** 2019-04-25

**Authors:** Heba H. Mostafa, Samantha M. Taffner, Jun Wang, Adel Malek, Dwight J. Hardy, Nicole D. Pecora

**Affiliations:** a Department of Pathology and Laboratory Medicine, University of Rochester Medical Center, Rochester, New York, USA; b Department of Microbiology and Immunology, University of Rochester Medical Center, Rochester, New York, USA; University of Arizona

## Abstract

The genome sequence of a Facklamia hominis strain isolated from the urine of a patient with acute cystitis and sepsis is reported. The genome contains *ermB* and *tet*(M) genes, consistent with the isolate’s phenotypic resistance to macrolides and tetracycline.

## ANNOUNCEMENT

Facklamia species are infrequently isolated Gram-positive cocci that have rarely been reported in association with human infections, including sepsis, genitourinary infection, wound infection, scapula abscess, prosthetic joint infection, and chorioamnionitis (1–9). Members of the genus are alpha-hemolytic, catalase negative, and positive for leucine aminopeptidase (LAP) and l-pyrrolidonyl-β-naphthylamide. Four species have been isolated from human sources (F. hominis, F. sourekii, F. ignava, and F. languida), of which *F. hominis* appears the most common (4, 6, 8). Here, Facklamia hominis was isolated from the urinary tract of a 75-year-old male who presented to the emergency department (ED) with fever and hematuria and was admitted for sepsis. The patient underwent a transurethral prostate resection to treat benign prostatic hyperplasia 12 days prior. His symptoms improved after treatment with ampicillin-sulbactam and vancomycin.

The isolate was cultured on Trypticase soy–5% sheep blood agar and incubated for 24 hours under 5% carbon dioxide. Multiple colonies were collected for DNA extraction. DNA was extracted using the MagNA pure compact platform (Roche) and quantified with the QuantiFluor double-stranded DNA (dsDNA) system (Promega). DNA library preparation was performed using the Nextera XT DNA library prep kit (Illumina). Pair-end sequencing (2 × 300 bp) was performed with MiSeq reagent v3 kits (Illumina). Trimmomatic (v0.36) (10) was used to ensure high-quality reads with trim criteria of 5:20 for SLIDINGWINDOW and MINLEN of 100. *De novo* genome assembly was carried out by SPAdes (v3.11.1) (11). General genome features and annotations were obtained with Prokka (v1.12) (12) and RAST (vClassic RAST) (13), and the antibiotic resistance genes were confirmed with ResFinder (The Center for Genomic Epidemiology) (14). For software or servers used for data analysis, default parameters were used unless otherwise specified. Phenotypic resistance was assessed by the MIC method (Sensititre Streptococcus STP6F; Thermo Fisher).

The genome size was 1,950,525 bp (G+C content, 39%). The assembly was composed of 203 contigs with an *N*_50_ value of 104,730 bp. Mapping the genome of our isolate to the *F. hominis* strains ACS-120-V-Sch10 (GenBank accession number KE340333), CCUG 36813 (NCBI accession number NZ_JH932292), and UMB0111 (NCBI accession number NZ_PKHF00000000) showed coverage of 86.3%, 89.3%, and 90.1% to the respective genomes, with an average depth of 373 reads.

RAST (vClassic RAST) identified 1,771 open reading frames, most of which were assigned putative functions. A total of 382 (21.48%) open reading frames were annotated as hypothetical proteins. System category distribution by RAST showed 292 subsystems, primarily for protein and carbohydrate metabolism. An *ermB* gene (conferring macrolide resistance) was found upstream of a *tet*(M) gene (conferring tetracycline resistance). Three prophage regions were identified using PHAge Search Tool Enhanced Release (PHASTER) (15), of which 2 (PHAGE_Bacill_vB_BhaS_171 [GenBank accession number NC_030904] and PHAGE_Entero_phiFL4A [NC_013644]) were described as incomplete (score, <70%) and the third (PHAGE_Bacill_1 [NC_009737]) was questionable (score, 70% to 90%). To corroborate the genetic resistance findings, studies were performed to determine the MIC, which showed that the isolate is phenotypically resistant to erythromycin, azithromycin, and tetracycline (following the CLSI M100 recommendations for testing and interpretive criteria for *Streptococcus* spp. of the viridans group [4]).

### Data availability.

This whole-genome shotgun project has been deposited at DDBJ/ENA/GenBank under the accession number RYDT00000000. The version described in this paper is the first version, RYDT01000000. The raw reads were submitted to NCBI SRA under accession number PRJNA510568.
